# Underestimation of overweight weight status in children and adolescents aged 0‐19 years: A systematic review and meta‐analysis

**DOI:** 10.1002/osp4.531

**Published:** 2021-05-31

**Authors:** Abrar Alshahrani, Farag Shuweihdi, Judy Swift, Amanda Avery

**Affiliations:** ^1^ Division of Nutritional Sciences University of Nottingham Nottingham UK; ^2^ Leeds Institute of Health Sciences Faculty of Medicine Leeds University Leeds UK; ^3^ Division of Food, Nutrition and Dietetics University of Nottingham Nottingham UK

**Keywords:** child, healthcare professionals, overweight, parents, underestimation and perceptions, weight

## Abstract

**Background:**

Perceptions of children's weight status may be important in obesity prevention and treatment.

**Aims:**

This review identifies the prevalence of the underestimation of overweight status in children by parents/main carers, children, and healthcare professionals (HCP). The review critically synthesized both quantitative and qualitative evidence to explore the factors associated with this underestimation. The diverse methods used to assess this phenomenon are reported.

**Methods:**

Pooled effect sizes were calculated using random‐effects model. Published studies, up to 2020, were accessed using the following search engines: CINAHL, EMBASE, PUBMED, and Psych‐Info and including the “Cited by” and “Related Articles” functions. Hand‐searching was used to retrieve further articles. Publication language and location had no bearing on the nature of the included studies.

**Results:**

A total of 91 articles were included. In the quantitative studies, 55% (95% CI 49%–61%) of caregivers underestimated their child's level of overweight and obesity using a verbal scale and 47% (95% CI 36%–55%) using visual scales. Of the children studied, 34% (95% CI 25%–43%) underestimated their own level of overweight and obesity using both scales. In (*n* = 3) articles, HCPs reflected this misperception, but limited studies prevented meta‐analysis. Underestimation was associated with the child's age, gender, BMI and parental weight status, ethnicity and education. In the qualitative studies, parents/main carers of children with overweight and obesity described their child's weight in terms other than overweight, for example, “big boned,” “thick,” and “solid.”

**Conclusion:**

The results confirm the prevalence of underestimation of child overweight status across international studies. Understanding the factors which lead to this inaccuracy may help to improve communication within the therapeutic triad and facilitate the recognition and management of children's overweight status.

## 1 INTRODUCTION

Levels of childhood overweight and obesity continue to increase globally.^1^ Approximately 41 million children aged 0–5 years are classified as overweight or obese, while 340 million children aged 5–19 years can be categorized as overweight or obese.^2^ Childhood overweight is a highly complex condition with biological, behavioral, social, economic, environmental, and cultural causes.^3^ The widespread nature of childhood overweight is a cause for concern since it is associated with a range of negative medical and psychosocial effects, both short and long‐term.^4^

Changes in diet, physical activity levels and, more recently sedentary activity are internationally considered to underpin interventions for the management of both adult and childhood obesity.^5^ To enact behavior change it is necessary to achieve concordance which, in the case of childhood overweight and obesity, involves the therapeutic triad of the child (if sufficient cognitive capacity), their parents/main carers, and healthcare professionals (HCP) involved in their care. Effective weight control interventions for children are strongly informed by the degree to which the overweight and obesity perceptions of children, parents/main carers and HCP are accurate and consistent with one another.^6^ It is, therefore, essential that weight‐related interventions are applied using an understanding of and effective working with key stakeholders and their perceptions of child overweight status.^7^

It is generally accepted that a significant barrier to intervention is that parents underestimate their children's overweight status.^8^ Furthermore, there is some limited evidence that children themselves and HCP may also underestimate.^9^ In‐depth engagement and cooperation across the therapeutic triad is required for concordance—a dynamic which would be threatened by misaligned perceptions of the situation.^6^ The psychological resistance that sometimes arises in parents due to the perceived link between childhood obesity, stigma, and blame, may result in their denial of a HCP's diagnosis, which decreases their receptiveness to the proposed interventions.^10^

Data from several studies suggest that negative emotions like anger and shame may lead to resistance in the form of refusing to take advice or ignoring referral appointments. Previous research has demonstrated that HCP can perceive conversations around weight to be “difficult” and thus avoid raising the subject at all.^11^ Understanding how sociodemographic, anthropometric, and cognitive factors, that may influence underestimation, may help to pre‐empt these “difficult” conversations, facilitate the recognition of children's overweight status, unify the therapeutic triad and help to notify the improvement of future weight‐related communications.

Earlier reviews in this area focused on parental perceptions toward child weight and repeatedly demonstrated that parents tend to underestimate their child weight.^12^, ^13^, ^14^ The most recent meta‐analysis searched databases up to 2013, reporting that 50.7% of parents underestimate their children's weight if they are living with overweight or obesity.^5^ The current systematic review and meta‐analysis aimed to update our knowledge by statistically identifying the prevalence of the underestimation of children's overweight status in children aged ≤19 years old in a wider sample (parents/main carers, children themselves and HCP). In addition, this review critically synthesized both quantitative and qualitative evidence to extend our understanding by investigating the factors associated with this underestimation. The systematic review also examined the diversity of methods used to assess this phenomenon.

## 2 METHODOLOGY

Studies, including quantitative and qualitative data on parents/main carers, children, and healthcare professional's underestimation of child overweight status, were systematically collected using the following search engines: CINAHL, EMBASE, PUBMED, and Psych‐Info from 1 January 2000 to 30 December 2020. Studies eligible for inclusion were qualitative and quantitative research that included children aged ≤19 years. In addition, the studies were also eligible for inclusion if they assessed parents/main carers, children, and healthcare professional's perceptions of children's weight using both a verbal scale (e.g., asking participants to complete a forced‐choice rating scale whereby each response category was based on a medical weight standard) or a visual scale (e.g., selecting an image from a range of pictures or silhouettes [linked blind to BMI and weight status]) that best represented the child's current size. The purposefully inclusive search terms (there is no standard terminology used in the literature) were “parents/main carers, child, HCP, overweight, obesity, weight, and underestimations.” Boolean operators were used to group the terms (see Table 1), thereby facilitating a more wide‐ranging search. In addition, a reference list of the identified literature was then hand‐searched and the “Cited by” and “Related Articles” functions in the search databases were also used to retrieve further relevant articles. Publication language and location had no bearing on the nature of the included studies. The participants were the parents/main carers of children with overweight/obesity, children with overweight/obesity or HCP. It should be noted that data were only on parents/main carers' assessments of their own children and not assessments of other children. Similarly, the children's assessment were of themselves and not other children, whereas the HCP′ assessment was generalized. Exclusion criteria included studies that do not provide anthropometric measurements with which to compare perceptions, as well as studies focused on perceptions toward “underweight” or “normal weight” participants only.

**TABLE 1:** Search terms used

The following phrases were altered to meet the search requirements of each single database AND			
OR	OR	OR	OR
Parent*	Perception*	Weight	Child*
Carer*	Underestimation*	“OverWeight”	Adolescents*
Guardian*	Underdetection*	“BodyWeight”	Children
Caregiver*	Belief*	“OverWeight”	Infant*
Mother*	Recognition*	“BodyWeight”	Youth
Maternal	View*	Obese*	Healthcare
Father*	Attitude*		Professionals
Paternal parent*	Perspective*		Physicians
Family grandparent*			Healthcare provider

### 2.1 Systematic review registration

The protocol has been registered at the International Prospective Register of Systematic Reviews (PROSPERO); registration number: **CRD42018085823**.

### 2.2 Quality assessment

A modified Newcastle‐Ottawa Scale adapted for single use in a specific context^15^ was used to assess the quality of the 91 included studies. Following the authors' guidelines each study was assessed on the following criteria: methods used to select participants (i.e., sampling bias), methods used for controlling confounding (i.e., performance bias), statistical methodology (i.e., detection bias), and what the methods used to measure the outcome variables are (i.e., information bias). This scale measures the risk on a scale of 0 (high risk of bias) to 3 (low risk of bias) (see Table 2).

**TABLE 2:** Results for quality assessments

Authors	Methods used to select participants (sampling bias)	Adequate sample size for the outcome of interest (performance bias)	Methods used for controlling confounding variables (performance bias)	Appropriate statistical methods (detection bias)	Handling missing data (detection bias)	Methods of the outcome measure (information bias)	Objective assessment method of the outcomes (information bias)
Abbott et al.^16^	3 LR	3 LR	3 LR	3 LR	3 LR	2 MR	3 LR
Adams et al.^17^	0 HR	3 LR	2 MR	3 LR	3 LR	2 MR	3 LR
Akerman et al.^18^	3 LR	3 LR	3 LR	3 LR	2 MR	3 LR	3 LR
Aljunaibi et al.^19^	3 LR	3 LR	3 LR	3 LR	2 MR	3 LR	3 LR
Al‐Mohaimeed^20^	3 LR	3 LR	2 MR	3 LR	3 LR	3 LR	3 LR
Al‐Qaoud et al.^21^	2 MR	3 LR	3 LR	3 LR	3 LR	3 LR	3 LR
Arcan et al.^22^	3 LR	0 HR	3 LR	3 LR	3 LR	3 LR	3 LR
Baughcum et al.^23^	3 LR	3 LR	3 LR	3 LR	3 LR	3 LR	3 LR
Bossink‐Tuna et al.^24^	3 LR	3 LR	3 LR	3 LR	3 LR	3 LR	3 LR
Boutelle et al.^25^	3 LR	3 LR	3 LR	3 LR	3 LR	2 MR	3 LR
Brann^26^	2MR	0 HR	3 LR	3 LR	3 LR	3 LR	3 LR
Brannon et al.^27^	3 LR	3 LR	2 MR	3 LR	3 LR	2 MR	3 LR
Campbell et al.^28^	2 MR	0 HR	3 LR	3 LR	3 LR	3 LR	3 LR
Carnell et al.^29^	2 MR	3 LR	3 LR	3 LR	3 LR	2 MR	3 LR
Chaparro et al.^30^	2 MR	3 LR	3 LR	3 LR	3 LR	3 LR	3 LR
Chen et al.^31^	3 LR	3 LR	3 LR	3 LR	3 LR	3 LR	3 LR
Chaimovitz et al.^9^	3 LR	0 HR	3 LR	3 LR	3 LR	3 LR	3 LR
Crawford et al.^32^	3 LR	3 LR	3 LR	3 LR	3 LR	3 LR	3 LR
de Hoog et al.^33^	3 LR	3 LR	3 LR	3 LR	3 LR	3 LR	3 LR
de La et al.^23^	2 MR	0 HR	3 LR	3 LR	3 LR	3 LR	3 LR
Duarte et al.^34^	2 MR	3 LR	3 LR	3 LR	3 LR	3 LR	3 LR
Eckstein et al.^35^	3 LR	0 HR	3 LR	3 LR	3 LR	3 LR	3 LR
Eli et al.^36^ 2014	3 LR	3 LR	3 LR	3 LR	0 HR	3 LR	3 LR
Esenay et al.^37^	3 LR	3 LR	3 LR	3 LR	3 LR	3 LR	3 LR
Etelson et al.^38^	3 LR	3 LR	3 LR	3 LR	3 LR	3 LR	3 LR
Fisher et al.^36^	2 MR	3 LR	3 LR	3 LR	3 LR	3 LR	3 LR
Garrett‐Wright^39^	2 MR	0 HR	3 LR	3 LR	3 LR	3 LR	3 LR
Gauthier and Gance‐Cleveland^40^, 2016	2 MR	1 HR	3 LR	3 LR	3 LR	2 MR	3 LR
Genovesi et al.^41^	3 LR	3 LR	3 LR	3 LR	3 LR	3 LR	3 LR
Guevara‐Cruz et al.^42^	3 LR	3 LR	3 LR	3 LR	0 HR	3 LR	3 LR
Ha et al.^43^	2 MR	3 LR	3 LR	3 LR	0 HR	3 LR	3 LR
Hackie et al.^44^	2 MR	0 HR	3 LR	3 LR	3 LR	2 MR	3 LR
Hager et al.^45^	2 MR	3 LR	3 LR	3 LR	3 LR	3 LR	3 LR
Harnack et al.^46^	2 MR	3 LR	3 LR	3 LR	2 MR	3 LR	3 LR
He and Evans et al.^47^	2 MR	0 HR	2 MR	3 LR	3 LR	3 LR	3 LR
Hearst et al.^48^	2 MR	3 LR	3 LR	3 LR	3 LR	3 LR	3 LR
Hudson et al.^49^	2 MR	3 LR	2 MR	3 LR	3 LR	3 LR	3 LR
Jackson et al.^50^	3 LR	3 LR	2 MR	3 LR	3 LR	3 LR	3 LR
Jansen and Brug^51^	3 LR	3 LR	3 LR	3 LR	0 HR	3 LR	3 LR
Jones et al.^52^	2 MR	3 LR	3 LR	3 LR	3 LR	2 MR	3 LR
Júlíusson et al.^28^	3 LR	3 LR	3 LR	3 LR	3 LR	3 LR	3 LR
Kasemsup and Reicks^53^	2 MR	0 HR	3LR	3 LR	0 HR	3 LR	0 HR
Kaufman‐Shriqui et al.^54^	2 MR	0 HR	2 MR	3 LR	3 LR	3 LR	3 LR
Kersey et al.^55^	0 HR	3 LR	2 MR	3 LR	3 LR	3 LR	3 LR
Kroke et al.^56^	2 MR	0 HR	2 MR	3 LR	3 LR	2 MR	3 LR
Lampard et al.^57^	3 LR	3 LR	3 LR	3 LR	3 LR	3 LR	3 LR
Laraway et al.^58^	2 LR	3 LR	3 LR	3 LR	0 HR	2 MR	3 LR
Lazzeri et al.^59^	3 LR	3 LR	3 LR	3 LR	3 LR	3 LR	3 LR
Lopes et al.^60^	3 LR	3 LR	3 LR	3 LR	3 LR	3 LR	3 LR
Lydecker and Grilo^61^	2 MR	3 LR	0 HR	3 LR	3 LR	0 HR	0 HR
Linchey et al.^62^	0 HR	0 LR	3 LR	3 LR	3 LR	3 MR	3 LR
Molina de Faria et al.^63^	3 LR	3 LR	3 LR	3 LR	3 LR	3 LR	3 LR
Mamum et al.^64^	2 MR	3 LR	0 HR	3 LR	2 MR	3 LR	3 LR
Manios et al.^65^	3 LR	3 LR	2 MR	3 LR	3 LR	3 LR	3 LR
Mathieu et al.^66^	2 MR	3 LR	2 MR	3 LR	3 LR	3 LR	3 LR
Maximova et al.^67^	3 LR	3 LR	2 MR	3 LR	2 MR	3 LR	3 LR
Maynard et al.^68^	3 LR	3 LR	3 LR	3 LR	3 LR	2 MR	3 LR
McKee et al.^69^	3 LR	3 LR	3 LR	3 LR	3 LR	3 LR	3 LR
Miller et al.^70^	3 LR	3 LR	3 LR	3 LR	3 LR	3 LR	3 LR
Moore et al.^71^	3 LR	0 HR	3 LR	3 LR	3 LR	3 LR	3 LR
Moschonis et al.^72^	3 LR	3 LR	3 LR	3 LR	3 LR	3 LR	3 LR
Musaad et al.^73^	3 LR	3 LR	0 HR	3 LR	3 LR	3 LR	3 LR
Myers and Vargas^74^	3 LR	3 LR	3 LR	3 LR	3 LR	3 LR	3 LR
Perrin et al.^75^	2 MR	0 HR	3 LR	3 LR	3 LR	2 MR	3 LR
Petricevic et al.^76^	0 HR	3 LR	3 LR	3 LR	3 LR	3 LR	3 LR
Ra et al.^77^	2 MR	3 LR	2 MR	3 LR	0 HR	3 LR	3 LR
Redsell et al.^72^	0 HR	3 LR	3 LR	3 LR	3 LR	3 LR	3 LR
Rosas et al.^78^	2 MR	0 HR	2 MR	3 LR	3 LR	3 LR	3 LR
Regber et al.^79^	2 MR	3 LR	2 MR	3 LR	3 LR	2 MR	3 LR
Reifsnider et al.^80^	2 MR	0 HR	3 LR	3 LR	3 LR	2 MR	3 LR
Robinson and Sutin^81^	3 LR	3 LR	2 MR	3 LR	3 LR	3 LR	3 LR
Rudolph et al.^73^	3 LR	3 LR	3 LR	3 LR	3 LR	2 MR	3LR
Ruiter et al.^82^	0 LR	3 LR	3 LR	3 LR	0 HR	3 LR	0 HR
Rodrigues et al.^83^	3 LR	3 LR	3 LR	3 LR	3 LR	3 LR	3 LR
Shrewsbury et al.^84^	3 LR	3 LR	3 LR	3 LR	3 LR	2 MR	3 LR
Skelton et al.^85^	0 HR	0 HR	3 LR	3 LR	3 LR	2 MR	3 LR
Spurrier et al.^86^	0 HR	0 HR	3 LR	3 LR	3 LR	2 MR	0 HR
Tarasenko et al.^6^	3 LR	3LR	3 LR	3 LR	3 LR	2 MR	3 LR
Thompson et al.^87^	3 LR	3 LR	3 LR	3 LR	3 LR	3 LR	3 LR
Tschamler et al.^88^	2 MR	0 HR	3 LR	3 LR	3 LR	3 LR	3 LR
Twarog et al.^89^	3 LR	3 LR	2 MR	3 LR	3 LR	3 LR	3 LR
Vanhala et al.^43^	0 LR	3 LR	3 LR	3 LR	3 LR	3 LR	3 LR
Vuorela et al.^90^	3 LR	3 LR	2 MR	3 LR	2 MR	3 LR	3 LR
Vrijkotte et al.^91^	0 HR	3 LR	3 LR	0 HR	3 LR	3 LR	3 LR
Wald et al.^92^	0 HR	3 LR	3 LR	3 LR	3 LR	2 MR	3 LR
Webber et al.^93^	3 LR	3 LR	3 LR	3 LR	3 LR	2 MR	3 LR
Wen and Hui^93^	3 LR	3 LR	3 LR	3 LR	3 LR	2 MR	3 LR
West et al.^94^	0 HR	3 LR	3 LR	3 LR	3 LR	3 LR	0 HR
Wong et al.^95^	2 MR	3 LR	3 LR	3 LR	3 LR	3 LR	3 LR
Yao and Hillemeir.^96^	2 MR	0 HR	2 MR	3 LR	0 HR	3 LR	3 LR
Young‐Hyman et al.^97^	0 HR	3 LR	3 LR	3 LR	3 LR	3 LR	3 LR

*Note:* *0 HR (HIGH RISK), 1 and 2 MR (MODERATE RISK), 3LR (LOW RISK).

### 2.3 Data extraction

Table 3 shows the summary characteristics of 91 studies included in this review. Data was extracted based on the study characteristics. These included authors/date, country and ethnic background, total number of participants, age of children and adolescents, sampling and recruitment; setting, respondents who provided the data, the overweight and obesity criteria applied, percentage of underestimation, weight status; and factors associated with underestimation were also extracted. Given that part of the aim of this systematic review was to examine the diversity of methods used to evaluate underestimations of children's overweight status, data on the verbatim questions that were asked of participants, verbatim response options, stated justifications for questions and/or responses, whether the participants were aware of weight status before answering, and whether participants were told of weight status, were also extracted. For the qualitative studies, the data of the participants' estimations, thoughts and views of children's weights were synthesized, with particular attention to overweight and obesity to produce a narrative understanding of the phenomena of interest.

**TABLE 3:** Characteristics of the included studies

Author, country	Sample characteristics: Age Ethnicity No. of participants Caregivers	Sampling, recruitment and settings	Question/s and answer/s	Cut‐off applied	Weight status Verbal‐visual under‐estimation %	Factors associated with under‐estimation
Abbott et al.^16^ Australia	5–17 years, Urban and rural Queensland 3043 participants and NA	A random sample from schools	**Q1** “how would you describe your child’s current body weight?” **A1** “too thin,” “About right,” “Too fat”	IOTF	OWOB 24% Parents‐verbal 60% Children‐verbal 36%	Child’s gender, child’s BMI
Adams et al.^17^ USA	4–8 years Native American 366 participants Mothers, grandmothers, fathers, and other guardians	Advertisements were posted in newspaper, samples were from schools and other community health fair locations	**Q1** caregivers were asked if child is overweight	CDC pre 2010	OW 26% Parents‐verbal 85%	Child's BMI, Grandmother as caregiver
Akerman et al.^18^ USA	6–14 years, Caucasians, Hispanics, African americans 1205 participants, Mothers, fathers, and other legal guardians	Convenience sampling from 18 malls	**Q1** caregivers were asked to provided details of their child’s height and weight	CDC pre 2010	AROW 17% OW 25% Parents‐verbal 61%	NA
Aljunaibi et al.^19^ UAE	6–19 years NA 1440 participants Mothers	Convenience sampling from public schools	**Q1** “do you think that your child’s weight is” **A1**“Underweight,” “normal,” or “overweight?”	CDC pre 2010	OW 14.7% OB 18.9% Parents‐verbal 27%	NA
Al‐Mohaimeed^20^ KSA	6–10 years NA 601 participants NA	Multi‐stage random sampling from schools	**Q1** “do you think that your child is obese?” **Q2** “is the child’s weight normal?”	NA	OW and OB in girls 23% in boys 17.5% Parents‐verbal 65%	Child’s BMI
Al‐Qaoud et al.^21^ Kuwait	3–6 years NA 2329 participants Mothers	Kuwait nutrition surveillance system study, recruited from school	**Q1** parents were asked if a child is overweight **A1** yes/no	WHO	All children were categorized as OW Parents‐verbal 97%	NA
Arcan et al.^22^ USA	4–7–9 years American Indian 124 participants Mothers, grandmothers, fathers/step fathers, and other	Convenience sampling recruited from schools	**Q1** “how would you describe your kindergarten child’s weight?” **A1** “very underweight,” “slightly underweight,” “about the right weight,” “slightly overweight,” and “very overweight”	CDC post 2010	29% OWOB Parents‐verbal 33%	Maternal BMI
Baughcum et al.^98^ USA	2–5 years Hispanic‐white, non‐hispanic black, hispanic‐black, Asian or Pacific Islander and other 622 participants Mothers	Convenience sampling recruited from paediatric practices and Kentucky WIC program	**Q1** “I feel my child is” **A1** “very under‐weight,” “a little underweight,” “about the right weight,” “a little overweight,” or “very overweight”	CDC pre 2010	OW 16% Parents‐verbal 79%	Maternal education
Bossink‐Tuna et al.^24^ Netherlands	2–4 years Urban and rural 682 participants Mothers and fathers	Convenience sampling recruited from health care centre	**Q1** “I am worried my child is overweight right now” **Q2** “In comparison with other children his/her age’ with regard to weight” **A1** “dis‐agree a lot,” “disagree a little,” “no strong feelings either way,” “agree a little,” and agree a lot” **A2** “much thinner” to “much more overweight”	IOTF	OW 7.2% Parents‐verbal 62%	NA
Boutelle et al.^25^ USA	14 years White, black, Asian, Hispanic, and other mixed 755 participants Mothers	Convenience sampling from Eating among Teens (EAT) project	**Q1** how they would describe their adolescent’s current weight? **A1** “very underweight,” “somewhat underweight,” “about right,” “somewhat overweight,” or “very overweight”	CDC pre 2010	OW 16.5% AROW 19.9% Parents‐verbal 70%	Child’s BMI
Brann^26^ USA	8–10 years Caucasian 49 participants Mothers and fathers	Flyers, newspaper adverts	**Q1** adolescent were instructed to choose a picture from the figure drawings, resembling their present size and the size that they desire to be **A1** Collins’ Figure Drawings (CFD)^99^ **Q2** parents were asked to rate their child’s ‘present’ body size, using Collins’ Figure Drawings (CFD)^99^	CDC pre 2010	OWOB 11%	NA
Brannon et al.^27^ USA	3–12 years Hispanic or African American 241 participants NC	Convenience sampling recruited from paediatrics clinic	**Q1** 5‐point liker scale was used when extracting information from parents about their child’s size **A1** “too skinny,” “thin,” “just right,” “somewhat overweight,” and “overweight.”	CDC pre 2010	OWOB 30.3% Parents‐verbal 20%	Child’s BMI
Campbell et al.^28^ Australia	4 years 945 participants Mother and fathers	The PEAS (Parent education and Support) program) A community‐based cohort study recruited from maternal and child health centres	**Q1** “I am worried my child is overweight right now” **Q2** “In comparison with other children his/her age’ with regard to weight” **A1** “dis‐agree a lot,” “disagree a little,” “no strong feelings either way,” “agree a little,” and “agree a lot.” **A2** “much thinner” to “much more overweight”	IOTF	OW 17% OB 3% Parents‐verbal 90%	NA
Carnell et al.^29^ England	5–3 years NA 564 participants Mothers	Sample recruited from primary schools, nursery and reception classes	**Q1** “how would you describe your child’s weight at the moment?” **A1** “very underweight,” “underweight,” “normal,” “overweight” or “very overweight”	IOTF	OW 26.9% OB 8% Parents‐verbal 81%	Child’s BMI
Chaimovitz et al.^9^ Canada	5–3 years NA 182 participants Mothers, fathers, grandmother and physicians	Convenience sampling from Children’s hospital for following patients coming to the clinic for a gastrointestinal condition	**Q1** selecting a picture or using words. Physicians also followed the same procedure for categorising the body size of each patient **A1** “underweight,” “slightly underweight,” “average,” “slightly overweight,” and “overweight”	CDC post 2010	OW 7.7% OB 15.4% Parents‐verbal 44% visual 47% Children‐verbal 44% Visual 43% HCP‐verbal and visual 33%	NA
Chaparro et al.^30^ USA	2–5 years NA 1702 participants Mothers	PHFE (public health Foundation Enterprises), then the special supplemental nutrition program for women, children (PHFE to WIC) and infants; taken via a computer aided telephone interviewing procedure	**Q1** mothers were asked “do you consider your child to be” **A1** “overweight,” “underweight” or “about right weight for (his) (her) height?”	CDC post 2010	OW 15.0% OB 17.0% Parents‐verbal 90%	Maternal BMI, child’s birth weight
Chen et al.^31^ USA	8–15 years NA 2613 participants mothers	NHANES, a nationally representative and multistage probability sample and noninstitutionalised US population conducted by the National center for health statistics. Interviewed at home	**Q1** mothers were asked “do you consider your child to be**”** **A1** “overweight, underweight or about right weight for (his) (her) height?”	IOTF	17.4% OW 19.8% OB Parents‐verbal 25% Children ‐verbal 27%	NA
Crawford et al.^32^ Australia	5–6 years and 10–12 years Melbourne 1210 participants Mothers and fathers	Stratified random sampling from schools	**Q1** centred around five choices, parents were instructed to categorize their child’s weight during the investigation **A1** “markedly underweight”; “underweight”; “average,” “overweight”; “markedly overweight.”	IOTF	In children aged 5–6, 23% were OWOB in children aged 10–12, 29% were OWOB Parents‐verbal 20%	Child’s age
de Hoog et al.^33^ Netherlands	5–7 years Dutch, native African descent Turkish, Moroccan and “other” 2769 participants Mothers and fathers	Follow‐up study group known as ABCD (Amsterdam Born child and their development). Sample recruited after their gynaecological visit	**Q1** “I feel my child’s weight is” **A1** “much too low, too low, normal, too high or much too high.”	IOTF	Dutch children OWOB 7.4% Turkish children OWOB 30.6% Moroccan children OWOB 28.0% Parents‐verbal 79%	Maternal educational, Immigrant generation
De La O et al.^23^ USA	5–12 years 10–12 years Non‐hispanic and white 576 participants Mothers or fathers	Connivance sampling from schools	**Q1** parents were asked to report their child’s weight **A1** “extremely overweight,” “somewhat overweight,” “about right,” “somewhat underweight,” or “extremely underweight”	CDC Post‐2010	OWOB 16% Parents‐verbal 75%	Child’s gender
Duarte et al.^34^ Brazil	12–36 months NA 135 participants Mothers and fathers	Where the connivance sampling from primary health care facilities	**Q1** “which picture looks most like your child?” Toddler silhouette scale (TSS)	WHO, 1995	AROW 23.7% OW 9.7% Parents‐visual 61%	Child’s BMI
Eckstein et al.^35^ USA	2–7 years Black, hispanic and white 223 participants Mothers	Consecutive sampling from paediatric practices	**Q1** “I feel my child is” **Q2** “did your child’s doctor ever tell you that your child is gaining weight too fast or is overweight?” **Q3** We also sked the participants to mark the drawing that looked the most like their child **A1** “underweight,” “a little underweight,” “about the right weight,” “a little overweight,” or “overweight” **A2** “yes,” “no,” or “I don’t know” **A3** Collins’ Figure Drawings (CFD)^99^	CDC pre 2010	AROW 19% OW 20% Parents‐verbal 64% visual 30%	Child’s age
Eli et al.^100^ USA	5–3 years Caucasian 56 participants NA	Posts in (job seekers website) and in local newspapers Parents were interviewed at the Oregon social learning centre	**Q1** “what do you think about your child’s (or grandchild’s weight)?” **Q2** In comparison to the child’s siblings, cousins and other children	WHO 2000	56% OWOB	NA
Esenay et al.^37^ Turkey	5–6 years Turkish mothers 1525 participants Mothers	Connivance sampling from public preschools	**Q1** “I am concerned my child is overweight right now” **Q2** “compared with peers,” with regards to weight **A1** a five‐point Likert scale (ranging from “disagree a lot” to “agree a lot”) **A2** “much thinner to much more overweight”	Turkish Children’s growth curve	OWOB 25.4% Parents‐verbal 73%	NA
Etelson et al.^38^ USA	4–8 years NR 83 participants Parents not specified	Convenience sampling from assessing the appointment log	**Q1** The method used to find out how parents viewed their child’s weight, was to ask them to draw a spot on a line	CDC	OW 23% Parents‐verbal 59%	NA
Fisher et al.^38^ Australia	4–8 years Australia (living in rural north western new south Wales) 946 participants Caregivers (not specified)	Stratified sampling from public primary schools in the NEAHS in 2002	**Q1** caregivers were asked to estimate their child’s weight **A1** “very underweight,” “underweight,” “normal weight,” “overweight,” and “very overweight”	IOTF	OW 13% OB 9% Parents‐verbal 56%	Child’s gender
Garrett‐Wright^39^ USA	2–5 years Caucasian, hispanic, African American and others 120 participants Mothers and fathers	Researchers approached volunteers for the study in clinics. Researchers used a convenience sampling method	**Q1** mothers were asked to give an account of their child’s weight for each phase of their life: Your child during the first year of life, as a toddler, as a pre‐schooler, as kindergarten, from 3rd through 5th grade, from 6th through 8th grade’ **A1** “markedly underweight Underweight, normal, overweight, markedly overweight”	CDC pre 2010	OW 5.8% Parents‐verbal 25%	Health literacy
Gauthier and Gance‐Clevelan^40^ USA	2–5 years Hispanic 83 participants Mothers	Convenience sampling from the head start program	**Q1** “I feel my child is” **Q2** mothers were asked to choose the drawing (linear) that looks the most like your child **A1** “very underweight, underweight, normal weight, overweight and very overweight” **A2 (**Eckstein and colleagues, 2006)^35^	CDC post 2010	OWOB 50.5% Parents‐verbal 50%	Maternal BMI
Genovesi et al.^41^ Italian Milan	4–10 years NR 569 participants Mothers	Healthcare facility	**Q1** “how would you evaluate your body weight?” **A1** “very underweight,” “underweight,” “about the right weight,” “overweight,” and “very overweight”	IOTF	OW 23.9% OB 11.2% Parents‐verbal 28%	Maternal education
Guevara‐Cruz et al.^42^ Mexico	8 years NA 273 **participants** Mothers	Convenience sampling from children’s hospital	**Q1** “your children’s weight is” **Q2** “your children’s weight is” **A1** “normal not normal” **A2** “normal, overweight or obese.”	CDC post 2010	OW 74% OB 27% Parents‐verbal 38%	NA
Ha et al.^101^ South Korea (Seoul)	6–7 years NA 340 participants Mothers	Six public schools recruited the mums of the children, who all had IDs	**Q1** “do you consider your child to be” **A1** “overweight,” “just right,” or “too thin (underweight)”?	Korean CDC 2007	OW 69% Parents‐verbal 14%	Child’s gender
Hackie and Bowles^44^ USA	2–5 years Hispanic families (Southern Nevada culture) 38 participants mothers	Convenience sampling from healthcare unit	**Q1** “do you think your child is overweight?” **A1** YES/NO	CDC pre 2010	All participants were categorized as OW Parents‐verbal 61%	Child’s age Maternal education
Hager et al.^45^ USA	12–13 months White and hispanic 304 participants Mothers	In WIC/urban paediatric clinics	**Q1** “which picture looks most like your child?” A toddler silhouette scale	WHO	OW 29.2% AROW 13.9% OB 15.3% Parents‐visual 70%	Child BMI
Harnack et al.^46^ USA	2–5 years White hispanic 1178 participants Mothers and fathers	Schools	**Q1** “how would you classify your pre‐schooler’s weight?” **A1** “very underweight,” “underweight,” “average weight,” “overweight,” and “very overweight.”	CDC pre 2010	AROW 25.5% OW 12.6% Parents‐verbal 91%	NA
He and Evanset al.^47^ England	4–6 years White 84.8% non‐white 15.2% 770 Participants Mothers	Convenience sample from elementary schools	**Q1** “Compared with other children the same age, do you feel your child is?” **A1** “underweight,” “slightly underweight,” “about the right weight,” “overweight, or obese.”	CDC post 2010	OW 17.1% OB 13.5% Parents‐verbal 63%	NA
Hearst et al.^48^ USA	12–13 months USA Majority were white 375 participants Parent (not specified)	Sample aetiology childhood obesity study from school	**Q1** “do you consider your child who is enrolled in the study to be” **A1** “very underweight,” “slightly underweight,” “about the right weight,” “slightly overweight,” or “very overweight”	CDC post 2010	OWOB 32.4% Parents‐verbal 31%	Child’s gender Parental education
Hudson et al.^49^ USA	3–5 years African American 86 participants Mothers and fathers	One of the two major urban head start centres	**Q1** ask parents to categorize their child’s weight, giving them this selection of answers to select from: **A1** “just the right size; underweight; overweight”	CDC post 2010	AROW 18.8% OB 15.6% Parents‐verbal 87%	NA
Jackson et al.^50^ USA	35–70 months White, black and Asian 107 participants Mothers	Convenience sample from pre‐schools	**Q1** “do you consider your child to be” **A1** “overweight, underweight, or about average in weight”	NCHS 1977	OW 15.9% Parents‐verbal 94%	Child’s BMI maternal BMI
Jansen and Brug^51^ Netherlands	9–11 years Dutch, Surinam, Moroccan, Turkish, Cape Verdian and other 1819 participants Mothers and fathers and others	Sample Rotterdam Youth	**Q1** “is your child overweight?” **A1** “not true,” “somewhat true,” and “very true,”	IOTF	OW 21.4 OB 7.4% Parents‐verbal 80%	NA
Jones et al.^52^ England	6–8 years White British 1072 participants Mother, fathers, stepmother, stepfather, grandmother or grandfather	Schools and home visits	**Q1** “how would you describe your child’s weight at the moment?” **A1** “very underweight,” “underweight,” “normal,” “overweight,” “very overweight.”	IOTF	OWOB 23.7% Parents‐verbal 69%	NA
Júlíusson et al.^102^ Norway	2–19 years NA 3770 participants NA	Random sample from Bergen growth study	**Q1** “what do you think about your child’s weight status” **A1** “much too thin,” “A little too thin,” “normal,” “A little too overweight,” and “much too overweight.”	IOTF	OW 10.7% OB 1.9% Parents‐verbal 70%	Child’ age Child’s gender
Kasemsup and Reicks^53^ USA	2–5 years among 80 participants Mothers	Schools	**Q1** mothers were asked to give an account of their child’s weight for each phase of their life: Your child during the first year of life, as a toddler, as a pre‐schooler, as kindergarten, from 3rd through 5th grade, from 6th through 8th grade **A1** “markedly underweight Underweight, normal, overweight, markedly overweight”	CDC Pre‐2010	AROW/OW 43% Parents‐verbal 77%	NA
Kaufman et al.^54^ Israel	4–7 years Europe former Ethiopia and East Africa 462 participants Mothers	Convenience sampling from volunteers in low socio‐economic status preschools	**Q1** mothers were asked to report their child as: **A1** “thin, normal weight or overweight”	WHO	OWOB 29.8% Parents‐verbal 82%	Child’s BMI
Kersey et al.^55^ USA	2–5 years Mexican American parents (Latino populations) 359 participants Mothers and fathers	Convenience sample from community health centre	**Q1** parents were asked to look at the group of line drawings and choose the child that they feel looks the most like theirs	CDC pre 2010	OW 21% Parents‐verbal 65%	NA
Kroke et al.^56^ Germany	3 months–7 years NA 235 participants Mothers	Participants recruited from the Dortmund nutritional and anthropometric longitudinally designed study	**Q1** “how do you perceive the body weight of your child?” **A1** “just right,” “a little too low,” “much too low,” “a little too high,” “much too high.”	German reference curves (10th UW; 10–90 HW, 0.90th OW, 0.97th OB.)	27% OW Parents‐verbal 82%	NA
Lazzeri et al.^59^ Italy	5–15 years NA 3076 participants Mothers	School	**Q1** “In your opinion your child is …” **A1** “very thin, a little thin, normal, a little fat, fat and very fat.” **Q2** “which of these figures is most similar to your child?”	IOTF	OW 21.8% OB 7.9% Parents verbal 35% visual 25%	NA
Laraway et al.^58^ USA	6–27 months White Mothers and fathers	Convenience sample from paediatric office	**Q1** parents were asked to choose from the options, how they feel their child’s weight was **A1** “much too low, low, just right, high, or much too high”	Age‐ and sex‐specific growth curves underweight <5th; healthy weight 5–85th; overweight 85–95th; obesity >95th	OW 18% Parents‐verbal 70%	NA
Lampard et al.^57^	6–14 years NA 347 participant**s** Mothers or fathers	Health care facility and school	**Q1** parents were asked to select figure that best represented their child’s body size **Q2** parents were also asked if they considered their child to be **A2** “underweight,” “normal weight,” “slightly overweight,” “moderately overweight,” or “markedly overweight”	IOTF	OW 30% OB 12% Parents‐verbal 95%	NA
Lopes et al.^60^ Portugal	9–12 years Urban 499 urban children Mothers	A school‐based study	**Q1** “how would you describe your child’s weight at the moment?” **A1** “underweight,” “normal weight,” “overweight or obese”	IOTF	OW 25.5% OB 6.4% Parents‐verbal 61%	Maternal educational
Lydecker and Grilo^61^ USA	5–15 years White, black, Hispanic and Asian 1007 participants Mothers and fathers	The Mechanical Turk online website	**Q1** mothers were asked to give an account of their child’s weight for each phase of their life: Your child during the first year of life, as a toddler, as a pre‐schooler, as kindergarten, from 3rd through 5th grade, from 6th through 8th grade’ **A1** “markedly underweight Underweight, normal, overweight, markedly overweight”	IOTF	OW 17% Parents‐verbal 45%	Child’s gender Child’s age Maternal BMI Less fat talk about persons with obesity and their child
Linchey et al.^62^ USA	8–12 years White, black, Hispanic and Asian 1002 participants Mother/Stepmother Father/Stepfather	A randomized controlled trial from school	Parents were asked to classify their child’s weight as “very underweight,” “somewhat underweight,” “about the right weight,” “somewhat overweight,” or “very overweight”	CDC post 2010	OW 26% OB 30%	Parent’s weight status and ethnicity not associated
Molina de Faria et al.^63^ Brazil	7–10 years white and non‐white 1282 participants Mothers	Conglomerate sampling from schools	**Q1** to examine the Mother’s view of her child’s nutrition, a multiple‐choice test was composed **A1** ‘underweight, normal weight, overweight, obese’	IOTF	OW 21% Parents‐verbal 61%	Child’s gender Child’s ethnicity
Mamun et al.^64^ Australia	5–14 years Australian 2650 participants Mothers	The sample was from a Mater‐University study of pregnancy	**Q1** “do you think your child is” **A1** “very underweight,” “slightly underweight” “about the right weight”, “slightly overweight” or “very overweight.”	IOTF	OW 24% Parents‐verbal 40%	Child’s gender Maternal BMI Child dissatisfaction Dieting
Manios et al.^65^ Greece	2–5 years NA 2278 participants Mother and fathers	Random sampling from nurseries and day care centres	**Q1** “I feel my child’s weight is” **A1** “much higher than normal,” “higher than normal,” “normal,” “lower than normal” or “much lower than normal.”	CDC pre 2010	24–36 months UW/NW (24%) AROW (23%) OW (20%) 37–48 months UW/NW (45%) AROW (43%) OW (41%) 49–60 months UW/NW (31%) AROW (34%) OW (39%) Parents‐ verbal 54%	Child’s gender Maternal education Children engaging in physical activity
Mathieu et al.^66^ Canada	2–6 years Canadian 1125 participants Mother and fathers	The Quebec longitudinal study of child development	**Q1** “In your opinion, compared with other children the same age and for his/her height, would you say that your child…” **A1** “is thin/slim,” “is of normal weight” or “is overweight.”	CDC Post‐2010	OWOB 77% Parents‐ verbal 77%	Child’s BMI
Maximova et al.^67^ Canada	2–17 years (Canadian children) 3665 participants NA	The Quebec child and adolescent health and social survey from schools	**Q1** children and adolescents chose the figure (the Stunkard body rating scale), that they felt was the optimum match up to their present appearance	CDC pre 2010	OW 12.7% OB 8.8% Children‐visual 22%	Maternal and schoolmate BMI
Moschonis et al.^72^ Greece	9–13 years NA 1858 participants Mother, grandparents nanny or others	Random, multistage and stratified sampling from schools	**Q1** “according to your perception, what is the current height of your child?” **Q2** mothers were also asked to select the picture that looked the most similar to their child’s body shape **A1** “underweight,” “normal weight,” “overweight,” and “obese”	IOTF	OW 30% OB 11% Parents‐verbal 15% visual 41%	Child’s gender Primary caregiver other than mothers (Nanny) Maternal BMI and education
Maynard et al.^68^ USA	2–11 years Non‐Hispanic white, non‐Hispanic black, Mexican American 5500 participants Mothers	An in depth stratified, multistage probability cluster sample from the National health and nutrition examination survey	**Q1** “do you consider [name of child] to be” **A1** “overweight, underweight, or about the right weight?”	CDC pre 2010	AROW 12.4% OW 10.3% Parents‐verbal 32%	Child’s gender Child’s age Child’s BMI
McKee et al.^69^ USA	Kindergarteners to 12th graders White and black 14,808 participants Mothers or fathers	Random sample from public school	**Q1** “what would you say best describes [your child’s] weight?” **A1** “underweight, healthy weight, overweight, or obese”	CDC pre 2010	OW 38.4% Parents‐verbal 82%	Child’s BMI
Miller et al.^70^ New Zealand	3–8 years White, Maori, and Pacific 101 participants Mothers or fathers	A sample was taken from the nutritional investigation on bone and dental health	**Q1** parents were asked to categorize their child’s weight **A1** “score 1 underweight”; “score 2 normal‐weight”; “score 3 slightly overweight”; “score 4 overweight”	CDC pre 2010	AROW 31% OW 9% Parents‐verbal 86%	NA
Moore et al.^71^ Atlanta and Georgia	2–17 years African American 77 participants Mothers	Connivance sample was from waiting room of health care facility	**Q1** “I feel my child is” **Q2** “has your child ever been told by a doctor that he/she is at‐risk for overweight, overweight, or obese?” **A1** “underweight,” “normal weight,” “a little overweight,” or “overweight” **A2** yes/no	CDC post 2010	AROW 19.5% OW 22.1% Parents‐ verbal 19%	Absent of physician comment on weight
Musaad et al.^73^ USA	22–63 months**‐** Hispanic, non‐Hispanic Black,Non‐Hispanic White, Other 497 participants Mothers	Unequal probability sampling from health care facilities	**Q1** mothers were asked to give an a description of their child’s weight, for each phase of their life: “Your child as a pre‐schooler.” **A1** “markedly underweight Underweight, normal Overweight Markedly overweight.”	CDC post 2010	OW 18% Parents‐verbal 90%	NA
Myers and Vargas^74^ USA	2–5 years Hispanic 200 participants Mothers or fathers	Convenience sampling from WIC health centre or child health services	**Q1** “do you think your child is overweight”? **A1** Yes, No	CDC post 2010	All participants were categorized as OB Parents‐verbal 45%	NA
Perrin et al.^75^ USA	4–12 years Nnon‐hispanic, black/African American 115 participants Parents (not specified)	Health care facility	**Q1** parents were asked to describe their child’s weight using standard terminology **A1** “underweight,” “healthy weight,” “at risk for overweight,” and “overweight”	WHO	All children were categorized as OW Parents‐verbal 91%	NA
Petricevic et al.^76^ Croatia	6–7 years NA 1068 participants Mother, fathers or both	From three health services	**Q1** “do you consider your child to be” **A1** “underweight,” “of normal weight,” or “overweight”?	NA	OW 12.6% OB 10.2% Parents‐verbal 0.3%	Child’s BMI
Ra et al.^77^ Korea	3–6 years Korean 388 participants Mothers and teachers	By putting adverts on the Korean association of public kindergarten Teacher’s website	**Q1** each child was asked to choose the photo that best resembled their present look (“which picture looks most like you?”) **Q2** mother and teacher were asked to select: (a) The body shape that looks the most like their child (b) The body shape that is perfect in their eyes Collins’ Figure Drawings (CFD)^99^	IOTF	OW 7.2% OB 1.6% Parents‐verbal 42% Children Verbal 39%	Child’s and maternal BMI Weight satisfaction Teachers' education level perception of and satisfaction with child’s body weight, attitude toward obesity Body image
Redsell et al.^72^ England	1–11 months White British, White European, Non‐white 38 participants Mothers and fathers	The UK National child measurement Programme recruited from attending child health clinics	**Q1** “can you describe your baby’s size at birth and your thoughts about this?”	CDC pre 2010	NA	NA
Reifsnider et al.^80^ USA	3 years Hispanic 25 participants Mothers	Convenience sampling from two Head Start centers	**Q1** mothers were asked about their view about their child’s weight **A1** “much too underweight” “slightly too underweight,” “proper weight,” “slightly too overweight” And “much too overweight”	CDC Pre‐2010	OWOB 19% Parents‐verbal 19%	NA
Robinson and Sutin^81^ **Study 1** Australia (Australian families) **Study 2** Republic of Ireland, Europe	Study 1 (4–5 years to 14–15 years) study 2 (9–13 years) NA Study 1 *N* = 2823 Study 2 *N* = 5886 Mothers	Two longitudinal cohort studies	Study **1**/**Q1** “which of these best describes your child?” **Q2** “which picture looks most like your body shape?” Study **2**/**Q1** for mothers “which do you think your child is?” **Q2** for children/“how would you describe yourself”? **Study 1** **A1** “underweight,” “normal weight,” “somewhat overweight,” and “very overweight” **Study 2** **A1** “very underweight,” “moderately underweight,” “slightly underweight,” “about the right weight,” “somewhat overweight,” “moderately overweight,” and “very overweight.” **A2** “very skinny,” “a bit skinny,” “just the right size,” “a bit overweight,” and “very overweight.”	CDC pre 2010	OW 61.9% OWOB 30.6% Parents‐verbal 55%	NA
Regber et al.^79^ Belgium, Cyprus, Estonia, Germany, Hungary, Italy, Spain and Sweden	2–9 years NA 16,220 participants Parents	The identification and prevention of dietary and lifestyle induced health effects in children and infants (IDEFICS) study from Kindergartens or school	**Q1** use the five‐point Likert system to assess the parent’s view of their child’s weight “Much too underweight” “slightly too underweight,” “proper weight,” “slightly too overweight” And “much too overweight”	IOTF	OWOB 18% Parents‐verbal 63%	child’s BMI
Rosas et al.^78^ Mexico and California	5 years NA 314 participants Mothers	A longitudinal birth cohort study of pregnant women and their children	**Q1** mothers were asked to choose the figure that they see as matching their child’s present appearance the most accurately **A1** Collins’ Figure Drawings (CFD)	IOTF	AROW California 15.0% Mexico 8.3% OW California 41.7% Mexico 6.7% Parents‐visual 90%	NA
Rudolph et al.^103^ Germany	7–17 years NA 285 participants Mothers and fathers	Convenience sampling from paediatric pulmonary disease out‐patient clinic	**Q1** children and parents were asked **A1** “very underweight,” “underweight,” “normal,” “overweight,” “very overweight.”	WHO	OW 3.5% OB 17% Parental‐verbal 2.0%	NA
Rodrigues et al.^83^	7–10 years 619 mothers and 174 fathers	Cross sectional study design from extracurricular sport and obesity rates in Portuguese elementary school children	“How would you describe your child's weight at the moment? Possible answers were one of the following four choices: “My child's weight is a bit too little,” “My child's weight is OK,” “My child's weight is a bit too much,” and “My child's weight is way too much.”	IOTF	OW 16% OB 6% Parents‐verbal 31%	Child’s age, gender maternal weight status
Ruiter et al.^82^	2‐12 years NA 2009 (*n* = 8105), 2013 (*n* = 8844) and 2017 (*n* = 11,022) NA	Complex sampling design from a community‐based survey	**Q1** “what do you think of your child’s weight?” **A1** “extremely low,” “low,” “normal,” “heavy,” and “extremely heavy”	IOTF	OW 17% OB 4% Parents‐verbal 95%	Child’s age, gender, parental higher education
Shrewsbury et al.^84^ Australia	13‐years and 15 years European 626 participants Mothers	Longitudinal Nepean study from Nepean hospital	**Q1** “how would you classify your child’s weight now?” **A1** “markedly underweight,” “underweight,” “average,” “over‐weight,” or “markedly overweight”	IOTF	13 years OW (27%) 15 years OW (90%) Parents‐verbal 54%	Child’s BMI child’ gender
Skelton et al.^85^ USA	10–19 years and parents of children aged 4–20 years African American 44 participants NA	Convenience sampling from the inner‐city health fair	Children were asked **Q1** “do you think you are” **Q2** “do you think your weight is” **A1** “underweight (too skinny),” “normal weight,” “overweight (weigh too much)” **A2** “healthy,” “unhealthy,” “other”	CDC pre 2010	37% AROW (19%) OW (18%) Parents‐verbal 80% Children‐verbal 70%	NA
Spurrier et al.^86^ South Australia	NA NA 44 participants NA	Health care facility	**Q1** ask participants, whether the child was: **A1** “underweight, acceptable weight, overweight or obese,” based on a series of body images	CDC/NCHS 2000	NA GPs 72% and paediatricians 68%	NA
Tarasenko et al.^6^ USA	8–15 years Non‐hispanic white, non‐hispanic black, and hispanic 4691 participants Caregivers (not specified) and HCPs	Multi‐stage probability sample from the 2005–2010 NHANES	**Q1** caregivers were asked if they view their child as: **Q2** guardians were asked if “a doctor or health care professional ever told” them that their child is categorized as overweight. HCPs’ estimations were based on the parents’ report of being informed by HCP that the child was categorized as overweight **Q3** ask children and adolescents, if they view themselves as: **A1** “overweight,” “underweight,” or “about the right weight.” **A3** “fat” or “overweight,” “too thin,” or “about the right weight.”	CDC post 2010	OW 16.4% OB 19.3% Parents‐verbal 70% Children‐verbal 42% HCP verbal 41%	NA
Thompson et al.^87^ USA	3–20 months Low‐income African American 237 participants Mothers	WIC clinics or a longitudinal cohort of the infant care and risk of obesity study	**Q1** open‐ended questions explored mothers’ perceptions of healthy infant **Q2** whether their infants can be categorized as under/overweight **Q2** “my infant is fat right now” and “my infant is thin right now” **A2** strongly disagree to strongly agree scale	CDC pre 2010	OW 3 months 29.5% 6 months 27.1% 9 months 24.4% 12 months 23.8% 18 months 19.4%	NA
Tschamler et al.^88^ USA	18 m −9 years Hispanic, non‐hispanic, white, black 139 participants Mothers and fathers	Convenience sampling from healthcare facility	**Q1** “would you classify your child as” **A1** “very underweight,” “a little underweight,” “about right,” “a little overweight,” or “very overweight”	CDC Post‐2010	NW 69% OW 31% Parents‐verbal 46%	NA
Twarog et al.^89^ USA	6–15 years White, black, hispanic and others 1449 participants NA	NHANES 2005–2008 and 2009–2012. In homes interview	**Q1** “do you consider (SP) now to be?” **Q2** “how do you consider SP’s weight?” **A1/A2** “overweight,” “underweight,” or “just about the right weight”	IOTF	All children were categorized as OWOB Parents‐verbal 70%	Child’s gender Child’s Ethnicity
Vanhala et al.^43^ Finland	10 years NA 749 participants Mothers	Health care clinics	**Q1** My child is **A1** “underweight,” “normal weight,” “slightly overweight,” and “heavily overweight.”	IOTF	At 5 years OW 13.3% OB 22.2% At 11 years OW 20% OB 23% Parents‐verbal 57%	Child’s gender Child’s BMI
Vuorela et al.^90^ Finland	5–11 years NR 606 participants Mothers or fathers	Convenience sampling from healthcare facility	**Q1** “do you consider your child underweight, normal weight, overweight or obese?”	IOTF	At 5 years OW 13.3% OB 22.2% At 11 years OW 20% OB 23% Parents‐verbal 51% Children Verbal 39%	NA
Vrijkotte et al.^91^	5–12 years 4488 participants Dutch, Surinamese, Turkish, Moroccan or other Mothers	Follow‐up study group known as Amsterdam Born children and their development (ABCD)	Mothers were asked what they thought of their child’s weight status **A1** “way too heavy”; “too heavy,” “just right,” “too light,” “way too light”	IOTF	OW 9% at baseline and 10.8% at follow‐up Parents‐verbal 80%	Ethnicity, education
Wald et al.^92^ USA	3–12 years African American and Caucasian 612 participants Mothers	Convenience sample design from healthcare facility	**Q1**. “Do you think your child is overweight?” **A1** yes/no	CDC pre 2010	AROW 15%; OW 44% Parents‐verbal 58%	Child’s gender
Webber et al.^93^ England	7–9 years White, Non‐white, Black and mixed black, Asian and mixed Asian 405 participants Mothers	Schools	**Q1** “how would you describe your child’s weight at the moment” **A1** “very underweight, underweight, normal, overweight and very overweight”	IOTF	OW 12.2% OB 3.8% Parents‐verbal 55%	NA
Wen and Hui^104^ China	10–15 years Urban 2162 participants NA	Stratified random sampling from schools	**Q1** parents were asked to describe their children’s weights subjectively **A1** “very underweight, slightly underweight, normal, slightly overweight and very overweight” **Q2** for adolescents “which picture looks the most like how you look”? **A2** Collins’ Figure Drawings (CFD)^99^	WHO	OW 16.7% Parents‐verbal 40% Children visual 30%	Adolescents’ gender
West et al.^94^ USA	3–12 years African American 2508 participants NA	A multi‐stage stratified sampling from schools	**Q1** parents were asked if they considered their child weight to be **A1** “overweight, at‐risk‐of‐overweight, a healthy or normal weight, or underweight”	WHO,1995	AROW 17% OW 21% Parents‐verbal 60%	Child’s Ethnicity
Wong et al.^95^ Taiwan	4–6 years NA 699 participants Children, mothers, fathers, grandparents, aunts and teacher	A purposive sampling, from public and private kindergartens	For children **Q1** “which picture looks the most like you?” For mothers **Q2** “which picture looks the most like your child?” **A1/2** Collins’ Figure Drawings (CFD)^99^	NHANES (1990)	OW 14.3% OB 8.3% Parents‐visual 57%	NA
Yao and Hillemeir^96^ China	6–12 and 13–18 years NA 1265 participants Mothers	An ongoing, population‐based longitudinal survey in a clinical setting	For mothers **Q1** “do you think your child is?” For children **Q2** “do you think you are now?” **A1/2** “underweight,” “normal,” or “overweight?”	CDC post 2010	OW 27.5% Parents‐verbal 72% Children ‐verbal 69%	Child’s BMI Maternal weight
Young‐Hyman et al.^97^ USA	5–11 years African American 111 participants Mothers or fathers	A purposive sampling from health care facility	**Q1** “which best describes your child’s weight?” **A1** “underweight, just right, overweight, very overweight”	NHANES (1990)	OWOB 91% Parents‐verbal 21%	NA

*Note:* *OWOB, Overweight or Obese; OW, Overweight; OB, Obese; NHANES, The National Health and Nutrition Examination Survey; NA, Not Applicable; HCPs, Healthcare Professionals; WIC, Women, Infants and Children.

### 2.4 Statistical methods

After the extraction of quantitative data, the main effect sizes for the percentage of underestimation by parents/main carers and children using both visual and verbal scales were determined using STATA 14 and a meta‐analysis was performed using *R* program (There was insufficient data to consider underestimation in HCP). For each study, the effect size was calculated for the reported adjusted proportion of the participants who underestimated children's overweight weight status. A random‐effects meta‐analysis model was used in the study since the studies stem from different populations and design‐related heterogeneity.^105^ To test heterogeneity between‐studies, the *χ* ^2^ with a statistic *Q* was used to examine the null hypothesis that all studies have the same true effect,^36^ namely H0: *τ*_2_ = 0. Given that the *Q*‐test sometimes may fail to detect heterogeneity when the number of samples is small (i.e., less than 10),^106^ the statistics *I*_2_ was also used to assess the proportion of the observed heterogeneity,^107^ as it is not affected by sample size. *I*_2_ values range from 0% to 100% with three levels of *I*_2_: low, moderate, and high corresponding to values of 25%, 50%, and 75%.^106^ In addition, the 95% CI around the *I*_2_ statistic was also calculated to determine whether the heterogeneity is present. Overall, *τ*_2_, *Q*‐test and *I*_2_ were used in this research to decide whether the estimated effects are homogeneous. Thereafter, pooled effect sizes were estimated via random effects models, since the studies are based on different populations. To visualize the overall estimates of the study effects with corresponding confidence intervals, forest plots were used.^108^

Sensitivity analyses of the meta‐analyses were conducted using a range of visual approaches including difference in values (DFFITS), Cook's distances, studentized deleted residuals, Cov‐ratios, and estimates of heterogeneity. This identified some potentially outlying studies, but with a low impact on overall estimate sizes, so they were retained in the meta‐analyses. In evaluating heterogeneity among the studies, it was apparent that there was a significant influence in all data‐sets, therefore, random effects models were applied to all studies.

## 3 RESULTS

### 3.1 Literature search

**FIGURE 1:**
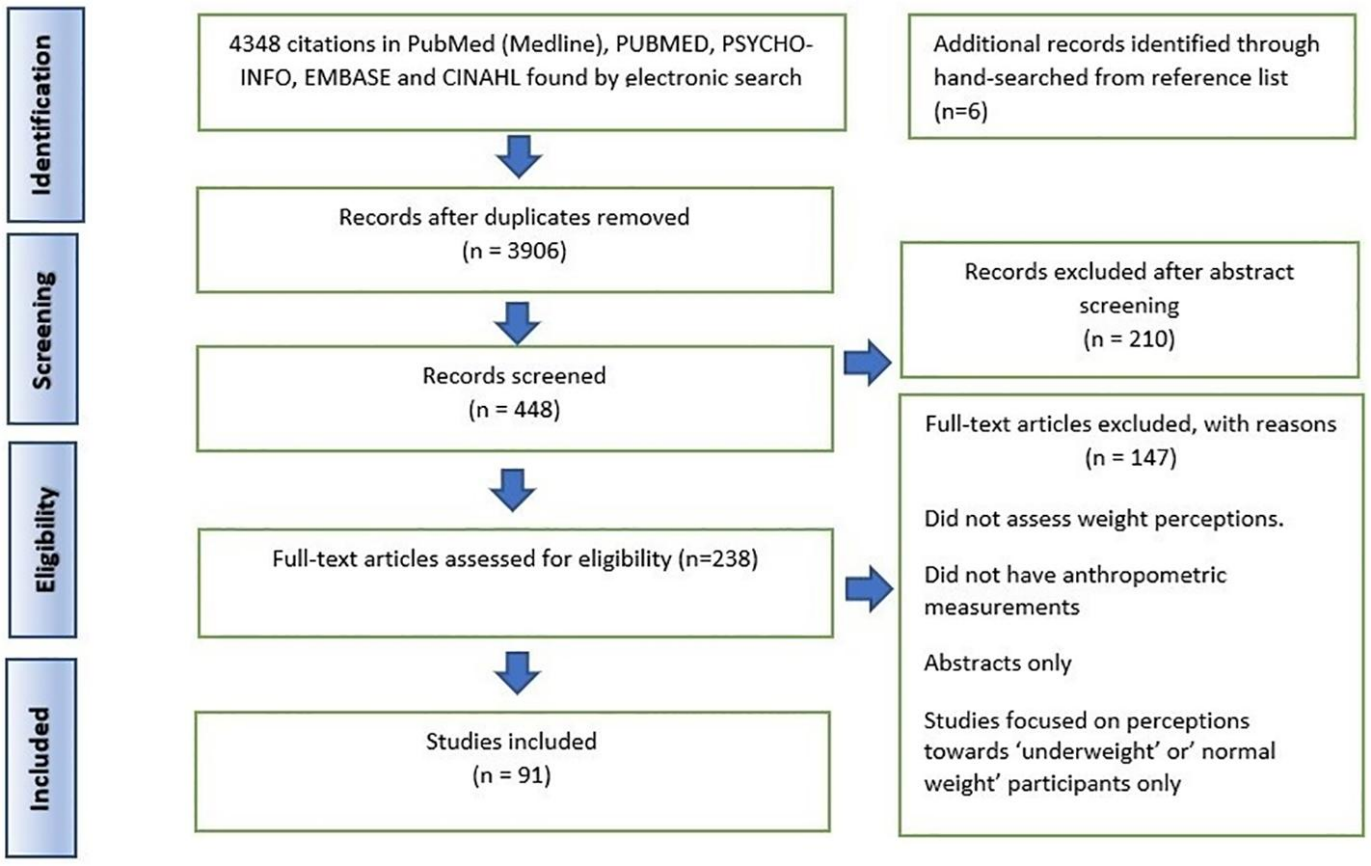
PRISMA flow diagram of the search strategy

A total of 4348 citations of potentially relevant references were identified with 3906 excluded after title screening. After an abstract and full text screening, a further 357 records were excluded. The reasons for the exclusions included lack of anthropometric measurements (*n* = 67), an absence of an assessment of weight perceptions (*n* = 58), and abstracts only (*n* = 22). After the exclusions, 91 studies fulfilling the inclusion criteria were included. Figure 1 depicts the results of the search strategy based on the PRISMA checklist.^109^

### 3.2 Study selection and characteristics

Table 3 shows the included studies (*n* = 91). The majority were quantitative, non‐experimental, cross‐sectional studies (*n* = 88), while a relatively small number (*n* = 3) were either qualitative only^72^, ^100^ or mixed methods.^87^ Verbal scales alone were used in the majority of the studies (*n* = 76), while visual scales alone were employed in eight studies, five of which applied both verbal and visual scaling.^9^, ^35^, ^59^, ^86^, ^110^ The studies were conducted across 20 different countries; 50% were conducted in European countries, 41% in the United States of America, 9% across Asia. All the included studies were in the English language and involved 24,774 participants‐children, parents/main carers and HCP. While (*n* = 29) studies did not report on ethnicity, the ethnic group that appeared with the greatest frequency across the studies were Hispanic, African American, White, Native American, and Asian. In terms of weight status, the majority of the studies included children ranging from all weight categories (*n* = 82), while for six studies, most children were classified as either overweight or obese.^17^, ^21^, ^34^, ^37^, ^38^, ^44^, ^45^, ^50^, ^56^, ^61^, ^67^, ^74^, ^75^, ^81^, ^89^, ^97^, ^99^, ^103^, ^111^, ^112^

### 3.3 Child weight status categorization

#### 3.3.1 Objective measures

Across the studies, all were based on weight to height ratios with different criteria for categorization to determine a child's weight status. The International Obesity Task Force's (IOTF) sex‐specific and age‐specific cut‐offs were used in (*n* = 30) studies, while (*n* = 24) studies classified children as at risk of overweight when their BMI is between 85th and 95th and overweight when their BMI is ≥to the 95th percentile (Centers for Disease Control and Prevention‐Pre 2010). The World Health Organization (WHO) growth standards were used in nine of the studies. Spurrier et al.^86^ used the 90th percentile as the cut‐off point for overweight, consistent with the CDC growth chart. The CDC post‐2010 was employed in several studies, which considers overweight to be a BMI between the 85th and 95th percentiles, while obesity is considered a BMI of greater than or equal to the 95th percentile (*n* = 17). Kroke et al.^56^ utilized German reference curves, classifying children as obese, where BMI was greater than or equal to the 90th percentile, while obesity was linked to the 97th percentile. Esenay et al.^37^ utilized the Turkish growth curves, which classify overweight based on the 85th and 95th percentiles and obesity as greater than or equal to the 95th percentile. Finally, Young‐Hyman et al.^97^ employed the National Centre Health Statistics, 1990 as the cut‐off point, which considers adolescents in the 95th to 99th percentiles to be classified as overweight, while those greater than the 99th percentile are classified as obese.

#### 3.3.2 Subjective measures

Across 80 of the studies, the participants verbally classified their children's or their own weight status with a forced‐choice rating scale, whereby participants described their weight status by selecting a category that was based on medicalized weight related standards. In 20 cases, a numerical Likert‐type scale was employed, in which a child's weight was considered along a spectrum, from very underweight to very overweight. Across the included studies, differing terms were used to describe overweight and obesity (e.g., “underweight” to “obese,” “underweight” to “overweight,” “somewhat thin” to “somewhat heavy,” “markedly underweight” to “markedly overweight” and “thin” to “fat”). One study relied on a visual analogue scale, where caregivers were requested to mark the spot on the line correlated with their child's weight status.^38^

In 13 studies, visual evaluations of sketches, silhouettes, photographs, and figures where used. In a group of silhouettes, participants were asked to select the figure that described their own child's or their own weight status. The majority used the validated pictorial instrument by Collin et al.^99^ (*n* = 6). Duarte et al.^34^ and Hager et al.^45^ used the Toddler Silhouette Scale (TSS) asking parents/carers, “Which picture looks most like your child?” Chaimovitz et al.^9^ compared parents', children's, and physicians' perceptions of children's weight using a body image scale to indicate the figure that most correctly describe the child's weight. Children alone were asked to select which silhouette looks most like their body shape in Robinson et al.^81^ and Maximova et al.^67^

Eckstein et al.^35^ examined a tool that involves gender‐specific and age‐range‐specific outlines of children to evaluate parental views of their child's body weight. When compared to written questions, images facilitated greater accuracy in the categorization of a child's weight based on their height. Furthermore, Lazzeri et al.^59^ reported that mothers tended to have more precise views of their children's nutritional status when selecting silhouettes corresponding to BMI, rather than when using multiple‐choice questions. Although Moschonis et al.^72^ reported that mothers' assessments of their children's weight status were more accurate when using verbal rather than visual classification tools, the study relied on outdated NCHS growth charts from 1979 which depicted only pre‐pubescent children. Chaimovitz et al.^9^ also employed verbal and visual scales for the assessment of perceived weight status, the intention being to safeguard against the influence of weight‐related terminologies used in the measure. The rate of weight status underestimation was comparable across the two scales, which is consistent with our meta‐analysis which provides very similar estimates of the extent of underestimation by parents/carers, regardless of the use of verbal or visual scales. The same process of underestimation of overweight and obesity was also found when HCP were asked whether each child was classified as underweight, having a healthy weight, overweight, or obese. Although there was insufficient data to perform a meta‐analysis, Spurrier et al.^86^ Chaimovitz et al.^9^ and Tarasenko, Rossen and Schoendorf^6^ all reported underestimation of overweight by a significant number of HCP. These studies included general practitioners (GPs) and pediatricians as well as physicians from hospitals, medical centers, and clinics. Among the reasons proposed for why HCP may underestimate children's overweight status is that, HCP do not take weight measurements or provide weight management guidance because their patients do not seem to be overweight or obese.^111^

Phenomenological methods were employed by Redsell et al.^72^ Eli et al.^100^ and Thompson et al.^87^ in order to gain insight into parents' perceptions of their children's weight status. These studies adopted qualitative research designs with semi‐structured interviews and focus groups. The results from the studies indicated that mothers conceptualized a child's weight differently and no participant held to the medical definition of childhood obesity which the researchers proposed. Instead a preschool child was considered to have a healthy weight if they could take part in a specific activity, appeared healthy, were happy, and were not bullied.^72^, ^87^, ^100^ Eli et al.^100^ reported that no participant described the weight status of preschool children as “obese” or “overweight” but used terms such as “chunky,” “stout,” “chubby,” “stocky,” “big boned,” “robust,” “solid,” and “pudgy.”

### 3.4 Methodological quality

Objective outcome measures were employed in almost every study (*n* = 89) except one where weight and height were reported by parents.^61^ In (*n* = 63) studies, it was not clear whether the experimental equipment was standardized and information was rarely given as to whether the objective child weight assessment process involved identical equipment across the entire sample. In the majority of studies (*n* = 80), data were not gathered as to whether participants already knew about the child's weight status. Weight classification methods, confounding variables, statistical analysis methods and the possibility of non‐respondent bias were identified in almost all studies as issues that could have reduced the methodological quality of the studies (see Table 2). Re; the diversity of the methods used to assess underestimation of overweight status, 75% of the included studies offer no explanation or justification for using specific terminology in question and/or responses in the verbal assessment method. The methodological quality ranged from high to low quality; however, the majority were relatively high in most of the studies (see Table 2). Therefore, the results seem to give a reliable estimate of the available data on this subject.

### 3.5 Meta‐analyses of underestimation

**FIGURE 2:**
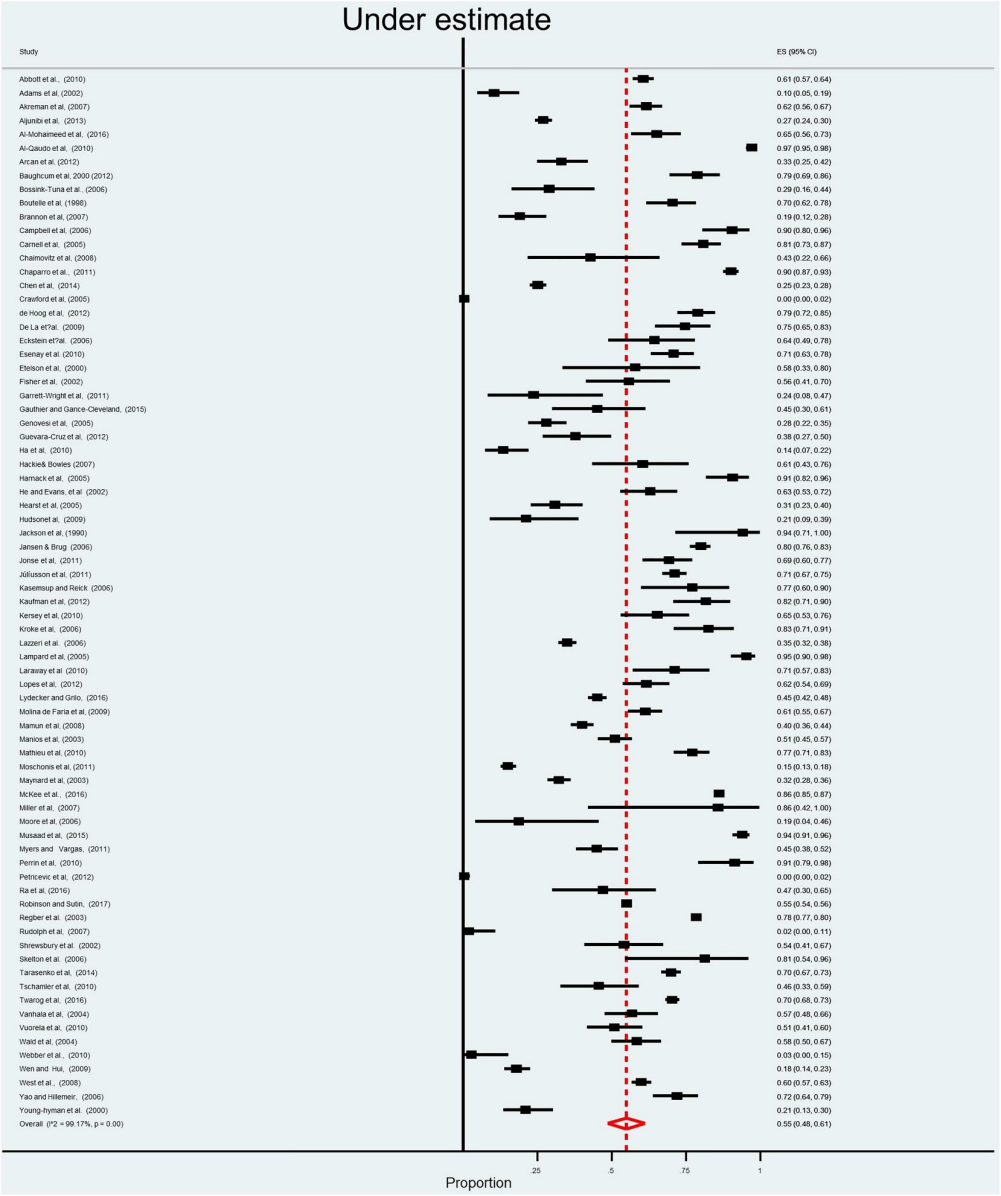
Forest plot for the summary of point estimates of effect sizes for caregivers' underestimation of their child's overweight status using verbal assessment scale

**FIGURE 3:**
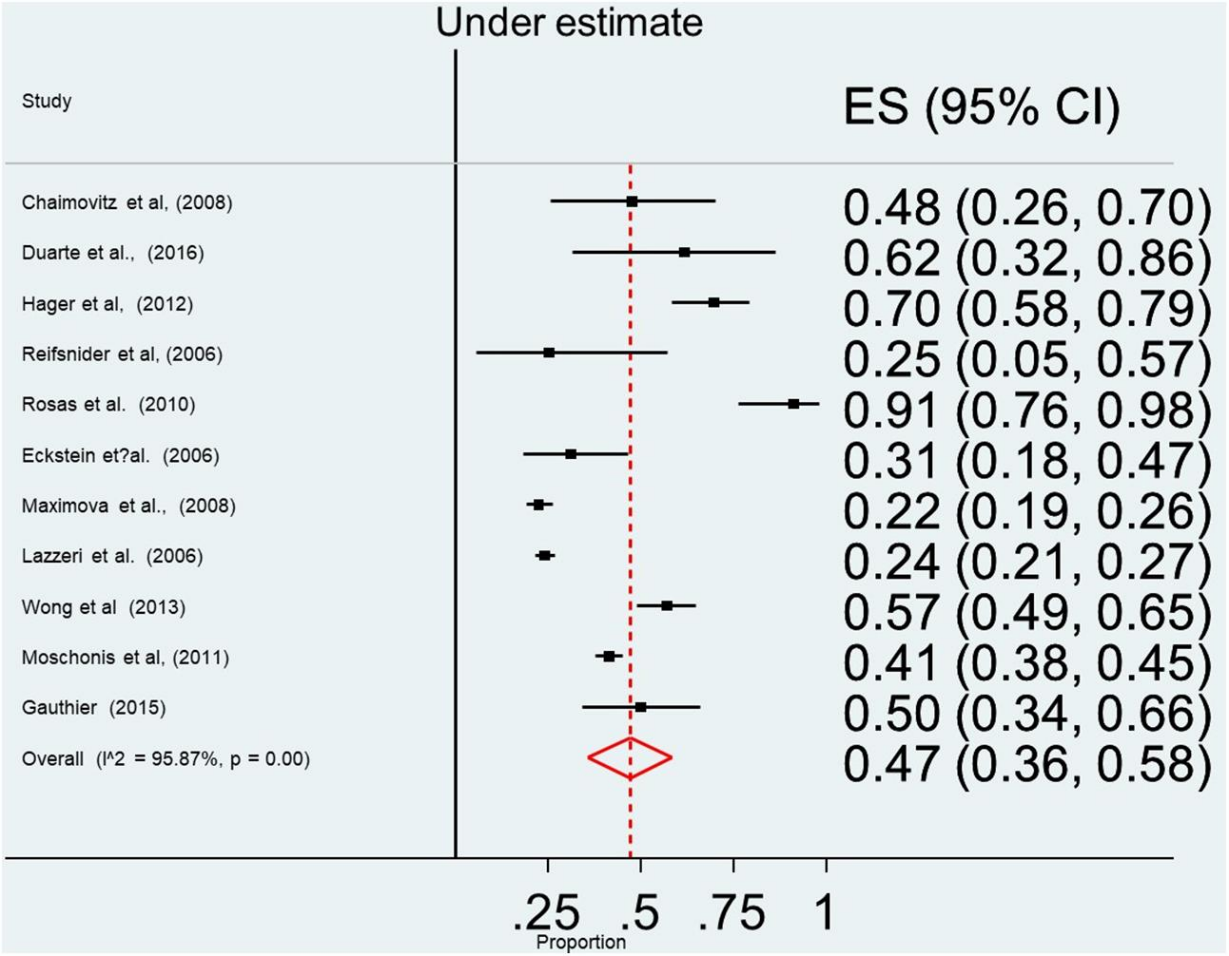
Forest plot for the summary of point estimates of effect sizes for caregivers' underestimation of overweight status is their children using visual assessment scale

**FIGURE 4:**
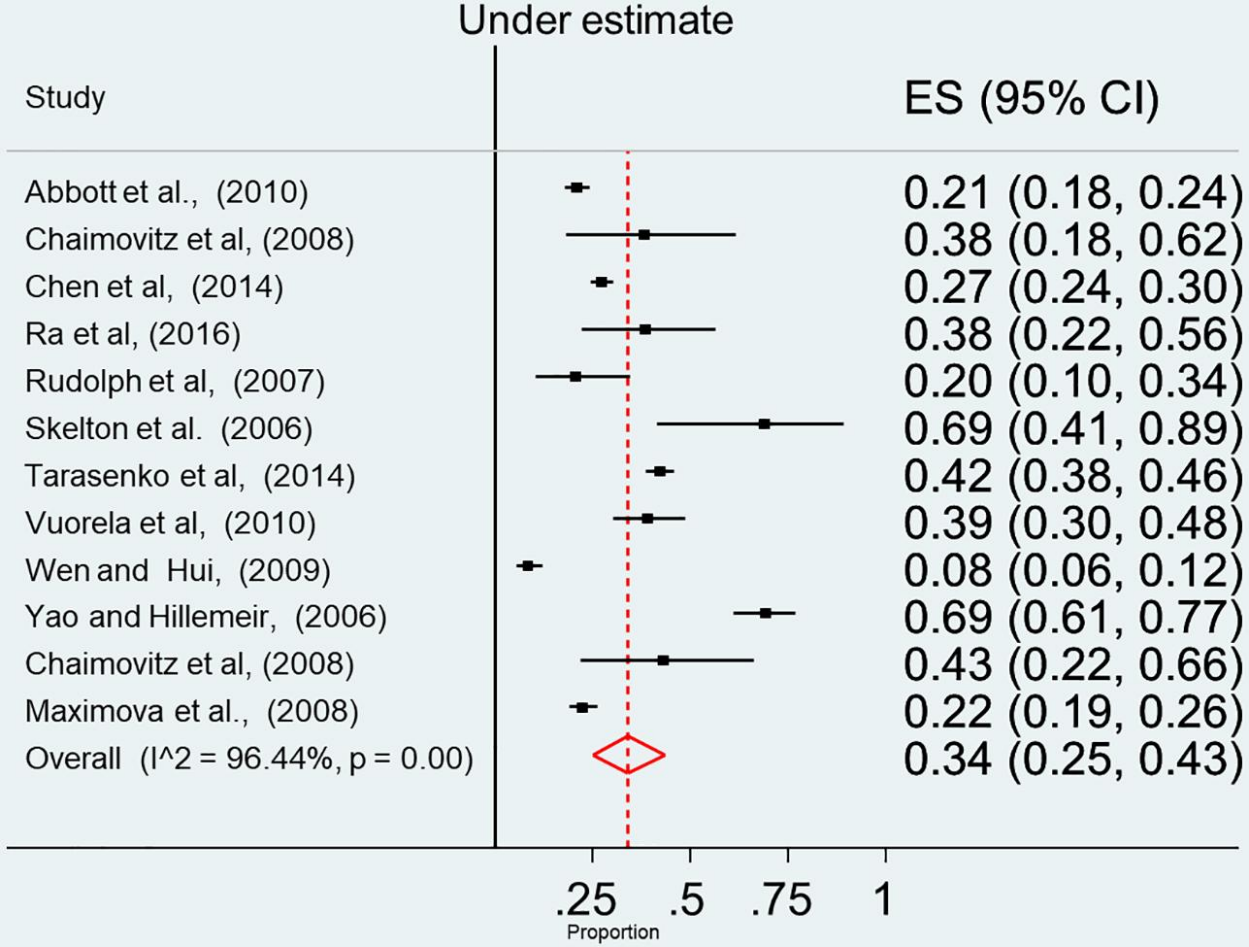
Forest plot for the summary of point estimates of effect sizes for children's underestimation of their overweight status

The main finding produced from the analysis of the quantitative studies is that the prevalence of underestimation of overweight is high among parents/main carers and children. When parents/main carers used a verbal scale (79 studies), 55% underestimated overweight and obesity (95% CI 48%–61%) (see Figure 2). Similarly, when using a visual scale (see Figure 3), the prevalence of underestimation by parents was 47% (95% CI 36%–55%). Children also tended to underestimate their weight (see Figure 4) with a prevalence estimate of 34% (95% CI 25%–43%). Although there was insufficient data to perform a meta‐analysis, Spurrier et al.^86^ Chaimovitz et al.^9^ and Tarasenko, Rossen and Schoendorf^6^ reported that underestimation of overweight also applied to a significant number of HCP with a range of (20%–60%).

The studies examined a number of factors that might contribute to inaccuracy in identifying children who were affected by overweight or obesity. These factors included the child's age where the overweight status of younger children was more likely to be underestimated than older children.^32^, ^35^, ^61^, ^68^, ^82^, ^83^ The child's gender was a factor that was found to be associated with the participants underestimation, as overweight and obesity in females was more accurately recognized than in males.^16^, ^43^, ^61^, ^68^, ^82^, ^83^, ^104^ Another factor was the child's current BMI. Children of higher BMIs were more likely to be correctly identified as overweight/obese,^16^, ^34^, ^43^, ^45^, ^47^, ^68^ suggesting that children close to the overweight but not obesity level were hardest for participants to classify. Maternal characteristics contributed to misidentification of children's overweight. Mothers with obesity were more likely to perceive their child's weight status incorrectly.^61^, ^67^, ^83^ Both child and parent ethnicity were also positively associated with the overall underestimation of the children's weight status in two studies.^63^, ^89^, ^91^ Parents with a lower education were more likely to underestimate their child's weight than relatively well educated parents.^33^, ^41^, ^62^, ^83^, ^91^, ^98^

Homogeneity assessment for point estimates of effect sizes of parental/main carers underestimation of their child's weight status in studies using the verbal assessment scale revealed high heterogeneity: *Q*‐statistic = 5218.9705, *p*‐value < 0.001. Similarly, homogeneity assessments, for point estimates of effect sizes of parental/main carers underestimation of overweight and obesity, in studies using the visual assessment scale, revealed high heterogeneity: *Q*‐statistic = 200.1098, *p*‐value < 0.001. In children based studies, homogeneity assessment for point estimates of effect sizes of the underestimation of overweight and obesity, also revealed high heterogeneity: *Q*‐statistic = 258.3483, *p*‐value < 0.001.

## 4 DISCUSSION

The current systematic review and meta‐analysis has statistically identified the prevalence of underestimation of child's overweight across a wide age range and across parents/main carers, children themselves and HCP. This review has shown that children's overweight status is frequently underestimated and is consistent with the earlier reviews conducted by Parry et al.^12^ Doolen et al.^13^ Rietmeijer‐Mentink et al.^14^ and Lundahl et al.^5^ However, it extends these works with a meta‐analysis that demonstrates that the majority of parents/main carers (55%, 95%CI 49%–61%) underestimated their children's level of overweight, while a substantial proportion of children underestimated their overweight status (34%, 95% CI 18%–54%). HCP shared this misperception despite the low number of studies preventing a meta‐analysis. This finding is in accordance with earlier literature, which shows that the underestimation of overweight is also very common in adults.^9^ The findings of this review are of concern if one accepts that alignment is required within the therapeutic triad for a trusting relationship and concordance.^9^ When one or more parties enters conversations minimizing or rejecting advice highlighting the need for intervention, it is reasonable to suggest that tensions might result unless time and consideration is paid to align beliefs.

To the authors' knowledge, this is the first systematic review of this topic which considers ways in which features of study design might affect the prevalence of the underestimation of overweight status, in addition to study quality. This systematic review has addressed studies which used both a verbal scale (e.g., asking participants to complete a forced‐choice rating scale whereby each response category was based on a medical weight standard) and/or a visual scale (e.g., selecting an image from a range of pictures or silhouettes [linked blind to BMI and weight status] that best represented the child current size) but considered them separate for the meta‐analyses. Across the literature, a wide range of terminology was employed to label response options in verbal scaling. These differing labels, even when the same criteria underpin the scaling, have the potential to be interpreted differently by participants. In addition, there is a sizeable evidence‐base demonstrating that overweight adjectives, including medical terms, can lead to negative emotions.^8^, ^9^, ^10^, ^13^, ^29^ In the qualitative studies, parents commonly describe their children in terms other than obese, such as “big boned,” “thick,” and “solid,” and demonstrate a strong desire to avoid labelling their child with medical terminology.^100^ Perceived blame, internalized stigma, and negativity may generate resistance to the language of the discussion, prevent accurate overweight status classification, and ultimately trigger reduced intention to engage in behavior change.^9^, ^10^ The effect of this on our results is that the studies, which utilized terminology associated with negative emotion, might under‐report the prevalence of overweight status and yet the majority of included studies (*n* = 66) neglected to describe the questionnaire development process and failed to justify the weight‐related terminologies used, even when in‐paper references to previous uses of the questionnaire were consulted (*n* = 12).

Visual scales avoid the need for labeled response options and the issues involved in selecting neutral terminology.^9^ However, the 13 studies using these methods are beset by the same variation in instruments (silhouettes, etc.) as the methodology utilizing verbal scales. Studies which allow direct comparison suggest that visual scales are more accurate than verbal scales.^59^ In view of this consideration, studies using pictoral assessment methods for the views of parents and children result in a slightly less underestimation of overweight/obesity (47%) compared to verbal scale (55%). This result may further suggest that parents and children/adolescents do recognize the weight status but do not verbally label it as overweight.^14^ This is perhaps because of the negative association with the word overweight and the stigma attached to people with obesity in society.^8^, ^68^ However, there is little evidence using pictorial assessment method in the current review to make informed conclusions on it.

Despite the diversity in the study design discussed above, it is noteworthy that such a large number of studies from across the world, have investigated this phenomenon and the meta‐analyses demonstrate such a clear effect. Any future studies investigating the prevalence of the underestimation of overweight status in children, particularly among caregivers, should be justified in the light of this. At this stage, more useful approaches will be applied in investigating family characteristics that are associated with overweight status underestimation as this review has suggested the influence of children's age (younger children),^32^, ^35^, ^61^, ^68^, ^82^, ^83^ gender (male),^16^, ^43^, ^61^, ^68^, ^82^, ^83^, ^104^ children's current BMI (higher BMI),^16^, ^34^, ^43^, ^45^, ^68^ and parental weight status (higher BMI),^61^, ^67^, ^83^ ethnicity^63^, ^89^, ^91^ and parental education levels (lower).^33^, ^41^, ^83^, ^91^, ^98^ The way in which an individual perceives another individual is informed by their relationship, the perceived individual's characteristics, and the perceiving individual's characteristics. This is further compounded by their beliefs, and experiences which, in turn, influence their attitudes, thoughts, and feelings toward the perceived.^14^ A clear knowledge of the factors which may lead to inaccuracy in assessing child overweight status may help to pre‐empt these “difficult” conversations, facilitate the recognition of children's overweight status and unify the therapeutic triad.

Jain et al.^113^ report two important findings: firstly, that the mothers of young children with overweight tended to assume that as their child aged, grew taller, and engaged in more physical activity, their child's weight status would improve and secondly, that continuous rises in weight and height were indicative of positive nutrition and parenting. Alongside this, Genovesi et al.^41^ reported that for some parents, overweight was considered a sign of good health. This may underpin why a child's overweight status is more likely to be underestimated by caregivers when the child is younger. Older children were found to be able to identify their own overweight status better compared to parents of younger children.^16^ This could be caused by their own perception of their weight compared to their peers. A number of studies included in this review report that participants underestimate overweight status less among female children when compared to male counterparts.^38^, ^43^, ^61^, ^68^, ^82^, ^83^, ^89^, ^104^ Drawing on a representative sample, one of the included studies reports that accurate identification of overweight status among mothers was three times higher for female children when compared to males.^68^ Similarly, Fisher, Fraser, and Alexander^112^ found that while 67% of parents of overweight males do not recognize this status, the proportion was 44% for females. According to Campbell et al.^28^ mothers have a higher sensitivity to female body image and weight considerations, while for overweight male children, their weight status may be perceived as physically advantageous. It is unfortunate that such a small proportion of the literature included male caregivers (*n* = 27) and for those that did, no direct comparisons were made.

A number of studies found that a child's BMI plays a significant role in affecting underestimations of overweight status; children with higher BMIs were more likely to be identified correctly as having an overweight status.^12^, ^16^, ^17^, ^34^, ^43^, ^45^, ^68^, ^84^ Therefore, children at the lower end of the overweight range (based on a BMI‐for‐age percentile) were more likely to be underestimated. This blurring at the overweight cut‐off level accounts, perhaps for norm‐comparison in a society where higher BMIs are more common.^114^

Maternal overweight weight status was a variable considered in a number of studies^61^, ^64^ with Moschonis et al.^110^ reporting that the underestimation of child overweight status was 1.44 times more likely among mothers with overweight compared to mothers without overweight status. Qualitative work by McPherson et al.^115^ addresses this by suggesting that because parents with a high BMI have been subjected to social stigma, they are more likely than underweight and healthy weight parents to want to safeguard their children from the provoking labels associated with overweight status. However, Linchey et al.^62^ found no association between parent weight status and underestimation. Despite the suggestion that lower levels of maternal education are associated with the underestimation of overweight in children^41^, ^44^, ^48^, ^62^, ^83^, ^91^, ^98^ there are some inconsistencies in the evidence by Adams et al.^17^ Carnell et al.^29^ and He and Evans^47^ suggesting no link between maternal educational levels. In contrast, Ruiter et al.^82^ found that the parents who had a high level of education were more likely to underestimate their child's overweight status. Ethnicity was also positively associated with the overall underestimation of child's weight status in two studies.^63^, ^89^, ^91^ Molina et al. 63 found white children were less likely to be underestimated than non‐white. The greater acceptance of larger body size in some cultures may account for these differences.^111^ In addition, parental underestimation of child's overweight and obesity has been reported to be particularly common among Hispanic individuals,^89^ an ethnic group with very high level of overweight and obesity prevalence and a likelihood to identify larger body sizes as being more normal.^111^ In the recent study by Linchey et al.^62^ parent race/ethnicity was not associated with underestimating child weight status. However, this study is limited by unequal ethnic groups (White, Hispanic, Asian, black), with a small percentage of African American (3.4%) which makes it difficult to fully examine the associations by ethnicity.

The study has found that there is much work to be done to elicit the relationship between parent/main caregiver, child and HCP's underestimation of weight status. A greater focus on the factors associated with HCP underestimation could provide a greater opportunity for a clearer understanding. Future studies could investigate additional factors such as the effect of male parents/main carers on the tendency to underestimate child overweight with multivariate study designs to allow a more complete and detailed description of any interactions between characteristics, and more consistent data collection methods with an emphasis on visual scaling.

This systematic review and meta‐analysis is associated with a number of strengths and limitations. A key strength is that it includes both quantitative and qualitative studies, which allows greater understanding. Over and above the existing knowledge in this area, the study sought to examine the perceptions of parents/carers, children, and HCP. This review includes participants from a range of socioeconomic and cultural backgrounds, with a wide age range of children (up to 19 years old) in both experimental and population settings. However, an important limitation of this systematic review has been the lack of a statistical examination of HCP' perceptions, which stems from the scarcity of relevant studies in the literature. Additionally, gender‐based disparities in the parental tendency to underestimate child overweight were not tested, since the majority of the included studies examined mother‐to‐child rather than father‐to‐child dyads. Lastly, the results could suffer from publication bias due to the fact that some of the unpublished studies were not included.

This systematic review's findings indicate that when the prevalence of underestimation of child weight status is acknowledged, viable interventions can be promoted which involve parents, children, and HCP. For the purpose of raising awareness among parents of children with overweight and obesity, it is necessary to ensure that HCP themselves have an adequate understanding of these conditions. This could be facilitated if HCP engage directly with the various ways in which children and their parents define overweight, and to gain insight into the long‐term consequential health factors of being categorized as overweight and obese.^116^

As emphasized by Puhl et al.^10^ the psychological resistance that sometimes arises in parents due to the perceived link between childhood obesity, stigma, and blame, may result in their denial of a healthcare professional's diagnosis, which decreases their receptiveness to proposed interventions. Hence, HCP should engage directly with concerns over stigma when discussing childhood overweight and obesity with parents, assuring them that they are not to blame for the condition, and that their participation in the cooperative management of their children's overweight condition represents a beneficial step forward. Additionally, HCP should refrain from engaging parents in such a way as to instill guilt or a sense of being judged. Lastly, sensitive framing of each discussion regarding child weight status should be a top priority.

## 5 CONCLUSION

The results of this wide‐reaching systematic review confirm that the underestimation of child overweight status is highly prevalent. Those that design child weight management interventions should expect that for many parents/main carers and children, identifying and problematizing their child's overweight status will be unexpected and requires extra consideration to align beliefs. It may also lead to negative emotions and resistance. Although understanding the factors which lead to inaccuracy in assessing child's overweight status may help to pre‐empt “difficult” conversations and facilitate the recognition of children's overweight status, much work is required. Future studies could usefully consider a wider range of parents/main carers, child and HCP characteristics, utilize multivariate study designs, and use more consistent data collection methods with an emphasis on visual scaling.

## CONFLICTS OF INTEREST

The authors declare no conflicts of interest.

## AUTHOR CONTRIBUTIONS

Abrar Alshahrani carried out the literature search, analyzed the data and wrote the manuscript. Farag Shuweihdi revised meta‐analysis. Judy Swift provided critical feedback to the manuscript. Amanda Avery provided critical feedback and helped shape the research, analysis and manuscript.
